# Supplementary material for: HP1γ Prevents Activation of the cGAS/STING Pathway by Preserving Nuclear Envelope and Genomic Integrity in Colon Adenocarcinoma Cells
Source: Int J Mol Sci. 2023 Apr 16;24(8):7347. doi: 10.3390/ijms24087347 (PMC10138453; doi:10.3390/ijms24087347)
Supplement: Supplementary file 1 [file ijms-24-07347-s001.zip › ijms-2280696-supplementary.pdf]

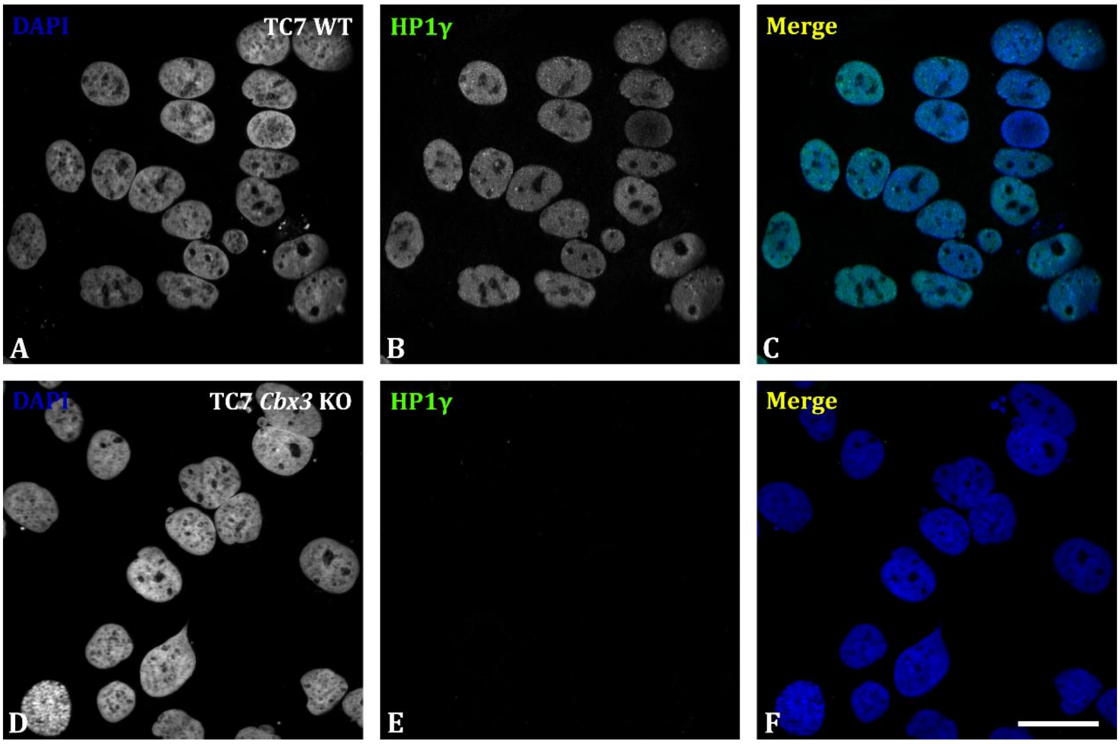

Supplementary Figure S1. Efficiency of *Cbx3* Knockdown in TC7 cells: (**A-F**) Immunofluorescence with anti- HP1γ antibody showing that the protein is abundant in the nucleus of WT cells (**B**) while it is undetectable in *Cbx3* KO cells (**E**). Images are representative of $n = 6$ clones (generated by 1 sgRNA) with 3 independent experiments/clone. Scale bar: 25 μm

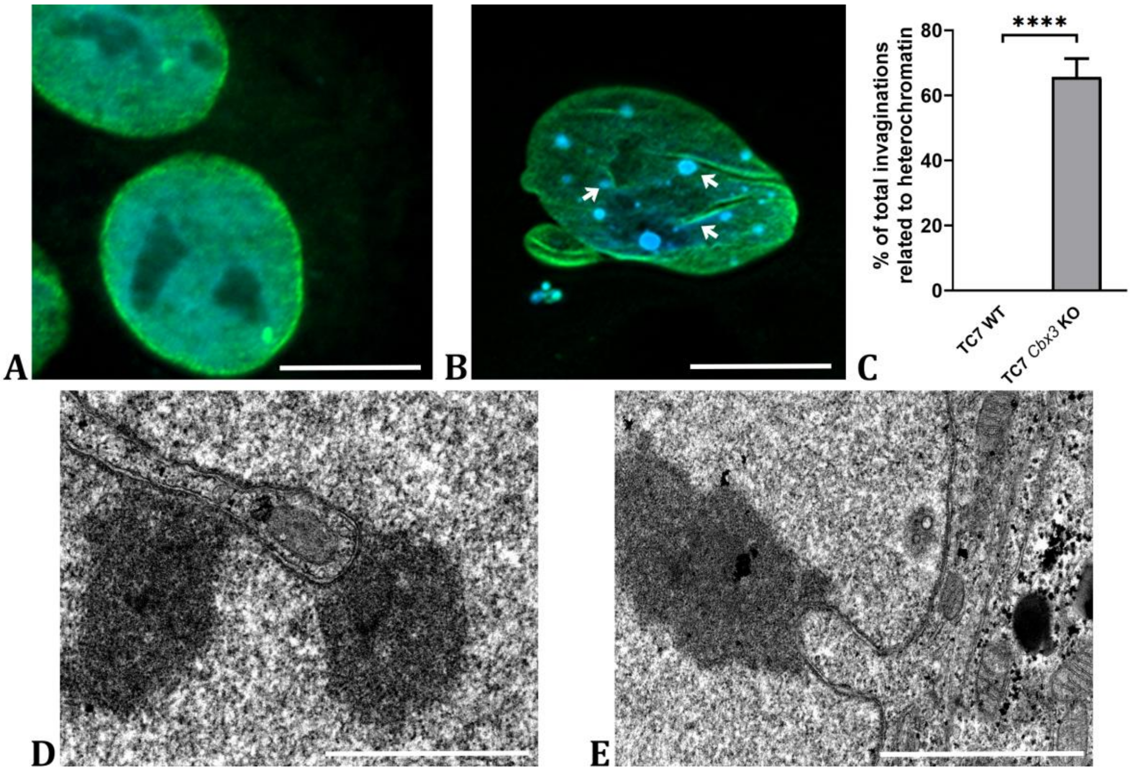

Supplementary Figure S2. Quantitative analysis of the number of nuclear envelope invaginations associated with heterochromatin. (**A**,**B**) Immunofluorescence of laminA/C showing envelope invaginations that tend to associate with heterochromatin foci, labeled by DAPI (**B**, arrows), While the nuclear envelope is shown to be uniform and without invaginations in TC7 WT cells (**A**). (**D**,**E**) Electron microscopy showing nuclear envelope invaginations in the TC7 *Cbx3* KO cells that associate with heterochromatin foci. (**C**) Quantitative analysis of the number of invaginations in which association with heterochromatin was observed. *n* = 6 clones (generated by 1 sgRNA), 3 independent experiments/clone (t-test and non-parametric, two-tailed P value). Scale bar: A,B = 25 µm; D,E = 1 µm.

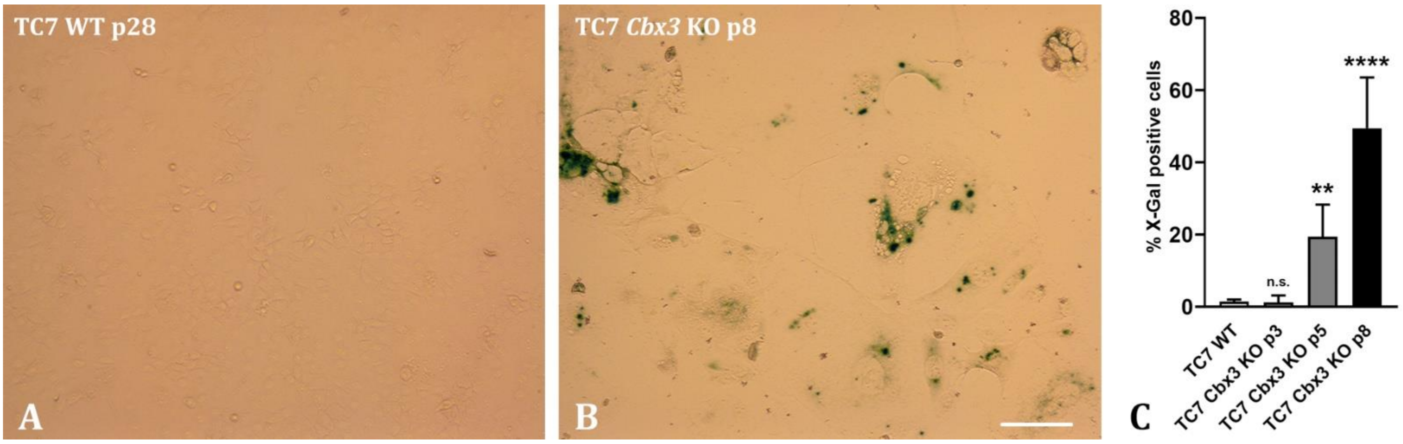

Supplementary Figure S3. Study of the viability of TC7 *Cbx3* KO cells by analyzing the incorporation of 5-bromo-4-chloro-3-indoyl β-D-galactopyranoside (X-Gal). X-Gal staining (greenish labeling) in (**A**) TC7 WT cells with no positive cells detectable whereas in *Cbx3* KO cells (**B**) a signal was detected, along with enlargement of the cell size (**C**) Quantitative analysis of the number of X-Gal positive cells in TC7 WT and TC7 *Cbx3* KO cells. WT cells show less than 1% of positive cells (passage 28), with a comparable level of X-Gal staining in TC7 *Cbx3* KO cells at passage 3. However, at passage 5, the percentage of positive cells increased considerably up to 20%, reaching a maximum of 50% at passage 8. At this point, cells lacking HP1γ stopped dividing. One-way ANOVA. ** $p < 0.01$; **** $p < 0.0001$. *n* = 6 clones (generated by 1 sgRNA), 3 independent experiments/clone. Scale bar: 50 µm.

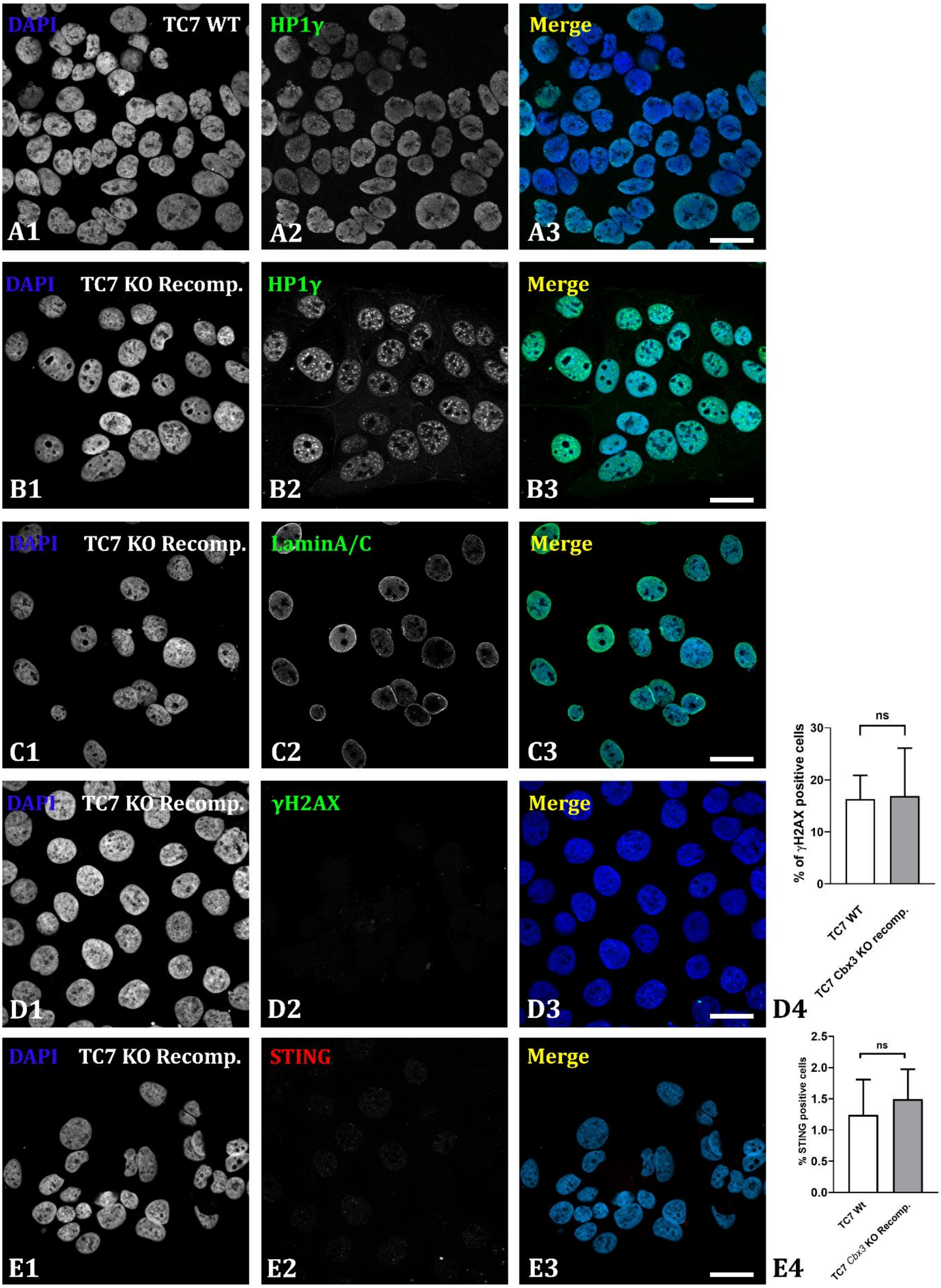

Supplementary Figure S4. Immunofluorescence analysis of the phenotype of TC7 *Cbx3* KO cells upon stable reintroduction of HP1γ expression: Immunofluorescences were performed as follow: (**A1**–**A3**) TC7 WT cells with Dapi/-HP1γ antibody; (**B1**–**E3**) TC7 *Cbx3* KO cells recomplemented with HP1γ with the corresponding antibody: (**B1**–**B3**) Dapi/anti-HP1γ, (**C1**–**C3**) Dapi/lamina/C, (**D1**–**D3**) Dapi/γH2AX. (**D4**) Quantification positive cells for γH2AX signal in WT and TC7 *Cbx3* KO cells recomplemented with HP1γ. (**E1/E3**) Dapi/STING. (**E4**) Quantification positive cells for STING signal in WT and TC7 *Cbx3* KO cells recomplemented with HP1γ. *n* = 3 clones (generated by 1 sgRNA), 3 independent experiments/clone (*t*-test and non-parametric, two-tailed P value). Scale bar: 25 μm.
